# The Braincase of *Eocaecilia micropodia* (Lissamphibia, Gymnophiona) and the Origin of Caecilians

**DOI:** 10.1371/journal.pone.0050743

**Published:** 2012-12-05

**Authors:** Hillary C. Maddin, Farish A. Jenkins, Jason S. Anderson

**Affiliations:** 1 Department of Organismic and Evolutionary Biology and Museum of Comparative Zoology, Harvard University, Cambridge, Massachusetts, United States of America; 2 Department of Comparative Biology and Experimental Medicine, University of Calgary, Calgary, Alberta, Canada; Raymond M. Alf Museum of Paleontology, United States of America

## Abstract

The scant fossil record of caecilians has obscured the origin and evolution of this lissamphibian group. *Eocaecilia micropodia* from the Lower Jurassic of North America remains the only stem-group caecilian with an almost complete skull preserved. However, this taxon has been controversial, engendering re-evaluation of traits considered to be plesiomorphic for extant caecilians. Both the validity of the placement of *E. micropodia* as a stem caecilian and estimates of the plesiomorphic condition of extant caecilians have been questioned. In order to address these issues, the braincase of *E. micropodia* was examined via micro-computed tomography. The braincase is considered to be a more reliable phylogenetic indicator than peripheral regions of the skull. These data reveal significant new information, including the possession of an ossified nasal septum, ossified anterior wall of the sphenethmoid, long anterolateral processes on the sphenethmoid, and paired olfactory nerve foramina, which are known only to occur in extant caecilians; the latter are possibly related to the evolution of the tentacle, a caecilian autapomorphy. A phylogenetic analysis that included 64 non-amniote taxa and 308 characters represents the first extensive test of the phylogenetic affinities of *E. micropodia.* The results place *E. micropodia* securely on the stem of extant caecilians, representing a clade within Temnospondyli that is the sister taxon to batrachians plus *Gerobatrachus*. Ancestral character state reconstruction confirms the braincase of *E. micropodia* to be largely representative of the plesiomorphic condition of extant caecilians. Additionally, the results refine the context within which the evolution of the caecilian form can be evaluated. The robust construction and pattern of the dermal skull of *E. micropodia* is interpreted as symplesiomorphic with advanced dissorophoid temnospondyls, rather than being autapomorphic in its robust construction. Together these data increase confidence in incorporating *E. micropodia* into discussions of caecilian evolution.

## Introduction

Of the three orders of living amphibians, caecilians remain the most poorly represented in the fossil record. Molecular evidence dates the divergence of the caecilian lineage from the frog-salamander lineage sometime during the Early Permian (299–270 m.y.a; [1]), or earlier [2]–[4], and the diversification of the majority of the currently recognized family-level clades by the end of the Mesozoic [5]. Despite this, only six accounts of crown-group fossil caecilians (Apoda) have been published to date (four Cenozoic and two Mesozoic forms [6]–[11]), five of which are based on specimens consisting only of vertebrae. However, as recognized members of the crown-group, these fossils do little to shed light on the origin of the caecilian form. Only two stem-group fossil caecilians (Gymnophiona) have been found (both Mesozoic [12], [13]), and only one of these, *Eocaecilia micropodia* from the Lower Jurassic of North America [12], [14], is represented by substantial parts of the cranial and postcranial skeleton. As the only well preserved stem-group caecilian, *E. micropodia* represents our best opportunity to understand the major morphological transformations that took place during the evolution of the highly specialized caecilian form, and of lissamphibians in general.

Wilkinson and Nussbaum [15] noted that the acceptance of *E. micropodia* as a member of the caecilian lineage had not been rigorously evaluated. Only a few phylogenetic analyses that include *E. micropodia* also include extant caecilians [16], [17], but more often *E. micropodia* is included to represent gymnophionans (e.g., [18]), thus precluding such an evaluation. Additionally, Gower and Wilkinson [5] have questioned, on the basis of several features, the validity of *E. micropodia* as representative of the plesiomorphic condition of caecilians, and Müller [19] raised the possibility that *E. micropodia* may actually occupy an alternative position within dissorophoid temnospondyl phylogeny. Central to these discussions has been the conspicuous observation that *E. micropodia* possesses a robust, stegokrotaphic skull with a closed temporal region which is in strong contrast to that of basal living caecilians where the temporal region is zygokrotaphic and widely open [12], [14]. Consideration of extant caecilian species has led to the hypothesis that an open temporal region is plesiomorphic for caecilians [19], [20]. Additionally, an open condition is present in basal members of all three groups of living amphibians [21], and recent work identifying homologous muscles passing through the temporal region and onto the dorsal surface of the skulls of frogs, salamanders, and caecilians supports the hypothesis that this condition is a synapomorphy of Lissamphibia [19], [22]. However, the presence of a closed skull in *E. micropodia* generates alternative hypotheses (reviewed in [19]). First, *E. micropodia* can be considered convergent with the stegokrotaphic condition seen in advanced caecilians due to similar fossorial habits. Or, second, the zygokrotaphic skulls of caecilians and batrachians can be considered convergent [23]. Resolution of these competing hypotheses is contingent upon the placement of *E. micropodia* in a phylogenetic context incorporating relevant fossil and extant taxa.

The braincase is a complex structure that has been demonstrated to be a more reliable indicator of phylogeny relative to the other parts of the skull in both amniote and non-amniote tetrapods [24]–[27]. This has been an especially valuable attribute when dealing with taxa that possess highly modified or functionally constrained skulls, as do caecilians. The tetrapod braincase appears very early in development, develops from a cartilaginous precursor, and is strongly shaped by the central nervous system [24]. As such, the braincase is morphologically constrained by these intrinsic factors and may be less prone to extrinsic influences such as environment and biomechanical adaptations. The braincase of caecilians, although somewhat modified in response to their burrowing habits, retains significant phylogenetic information capable of resolving genus-level relationships [28]. However, how deep within the caecilian lineage this potential of the braincase persists remains unknown.

The goals of the current study are: 1) to further investigate the morphology of the skull, particularly the braincase, of *E. micropodia*, using micro-computed tomography to explore whether the braincase reveals phylogenetic information; 2) to test hypotheses of affinities in a broad phylogenetic analysis including *E. micropodia,* within the greater context of non-amniote tetrapods; and 3) to use these data to further understand the origin and evolution of caecilian form, and that of lissamphibians in general.

## Materials and Methods

### Specimens

The holotype specimen of *Eocaecilia micropodia* (MNA V8066), consisting of a nearly complete skull and lower jaw, was subjected to micro-computed tomography (µCT) at the University of Texas CT facilities (Austin, Texas). The scan was performed at 210 kVp and 0.11 mA. Voxel resolution was 21.5 µm^3^. An additional specimen consisting of an isolated braincase belonging to *E. micropodia* (MNA V8063) was subjected to µCT in the SkyScan 1173 (Kontich, Belgium) at the University of Calgary. The scan was performed at 80 kVp and 60 µA, with a voxel resolution of 12.1 µm^3^.

### Visualization of Micro-computed Tomography

All scan data were down-sampled to a maximum of 512 pixels in any orientation, rendered as 8-bit greyscale TIFFs using the batch processing function in Photoshop CS2, and imported into Amira v.4 and v.5 (Visage Imaging, San Diego, CA) as a series of stacked images. The elements of the braincase were isolated by labelling structures using the LabelFields module, and visualized by applying the SurfaceGen and SurfaceView modules to the labelled data. The morphology of the braincase and stapes is described here based on the three-dimensional SurfaceView models generated from the µCT datasets.

### Phylogenetic Analysis

The character-taxon matrices of Anderson et al. [18] and Maddin et al. [28] were utilized in the current study, providing broad coverage of both fossil and extant taxa. The matrix of Anderson et al. [18] was modified as per Maddin and Anderson [16], with additional amendments made here (see Table S1 in supporting information ). Modifications to the matrix of Maddin et al. [28] include revised definitions of several characters that make them relevant to non-caecilian taxa, as well as 28 character exclusions due to redundancy with characters in Anderson et al. [18] or autapomorphy. These changes are indicated in the character list presented in Text S1 (see supporting information). The taxa included in Anderson et al. [18] were scored for the characters of Maddin et al. [28], where applicable. The taxa of Maddin et al. [28] were reduced to family-level taxa and scored as composites for all characters, some of which, as a result, are scored as multistate taxa. Additionally, five new characters and the stem salamander, *Karaurus* (not included in either previous matrix), were added. The terminal taxa “frogs” and “salamanders” were scored based on observations of the basal taxa *Ascaphus* (Redpath Museum 2184) and *Hynobius* (University of Alberta Museum of Zoology 3635). The resulting matrix consists of 64 taxa and 308 morphological characters, 274 of which are parsimony informative in the current analysis (see Dataset S1 in supporting information). The scores for *E. micropodia* included 24.1% missing data.

The matrix was analyzed in PAUP* v.4.0b10 [29]. *Acanthostega* was set as the outgroup for rooting. The heuristic search option was used, the tree bisection and reconnection (TBR) option was selected and the multiple trees (MulTrees) option was in effect. Multistate taxa were treated as polymorphic and all characters were unordered and equally weighted. Bootstrap support was determined using the full heuristic search option for 500 replicates in PAUP. Indices of goodness of fit of the character data to the topology (e.g., consistency index, retention index, rescaled consistency index and homoplasy index) were calculated in PAUP.

### Ancestral Character State Reconstructions

Because the braincase was the focus of the current study, ancestral character state reconstructions of the 34 caecilian braincase characters from Maddin et al. [28] were performed in order to assess whether or not the condition present in *Eocaecilia micropodia* is representative of the plesiomorphic condition of the braincase for Apoda. The state of each character at the base of Apoda was estimated using maximum parsimony and maximum likelihood (Mesquite v.2.72; [30]), and Bayesian inference (BayesTraits v.1.0; [31]). For the maximum parsimony and maximum likelihood approaches, the maximum clade credibility tree from the analysis of Maddin et al. [28] was used as the topology upon which character evolution was reconstructed. For the Bayesian estimation a sample of 1000 post-burn-in trees generated in the Bayesian analysis of phylogeny from Maddin et al. [28] were imported into BayesTraits, along with an input file consisting of the character states associated with each species for the thirty-four braincase and stapes characters. The Multistate and MCMC options were selected for the analyses. Each ancestral character state reconstruction analysis was run for 1 million iterations, at which point the harmonic means of the log likelihoods were observed to reach stationarity. The run was sampled every 1,000 iterations, after a burn-in period of 100,000 iterations. The rate of deviation parameter (ratedev) was adjusted to obtain a recommended level of acceptance (20–40%, in this case, using a ratedev of 5), and the reverse jump hyperprior was set to exponential on an interval from 0 to 30, as per the program’s recommendations. The Fossil Node function was applied to each character to test hypotheses of which state is more likely to occur at the node of interest, in this case the base of Apoda. This function ‘fossilizes’ the state at this node and indicates the probability of that state being present at that node, based on the distribution of states in the terminal taxa. State 0 was first fossilized, followed by state 1 (then state 2, etc., if applicable). To determine which state is more likely, a Bayes Factor test was applied. This was accomplished by subtracting the harmonic mean of the log likelihoods after 1 million iterations for state 1 from the harmonic mean of the likelihoods for state 0, and multiplying this value by 2 [31]. In general, positive values are taken as favouring the first model, but are not necessarily statistically robust. Values greater than 2 are considered to support the first model, values greater than 5 are considered to strongly support the first model, and values greater than 10 to very strongly support the first model [31]. The results of the three approaches were compared for congruence.

## Results

### Morphology of the Braincase of Eocaecilia Micropodia

**Figure 1:**
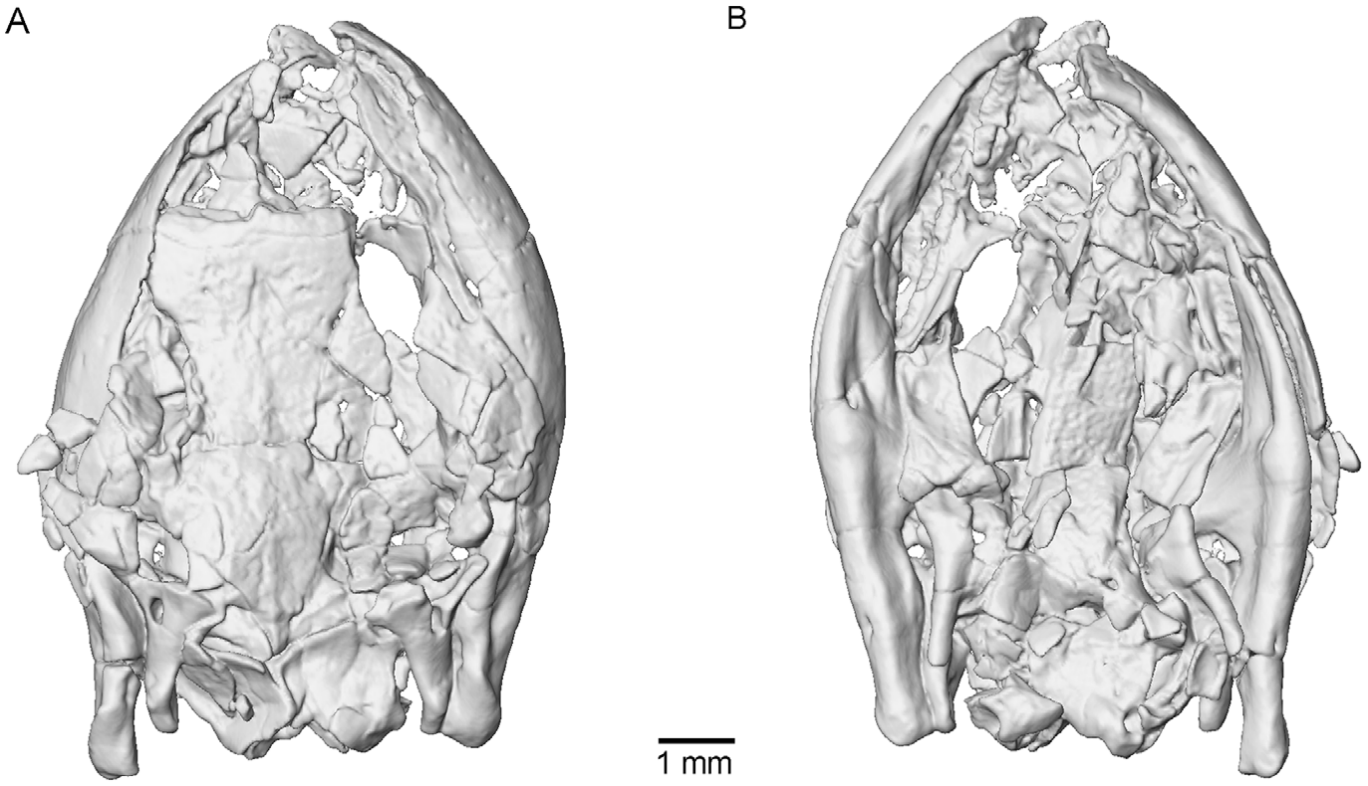
Volume rendering of the µCT data of the holotype of *Eocaecilia micropodia* (MNA V8066). A, dorsal view. B, ventral view.

**Figure 2:**
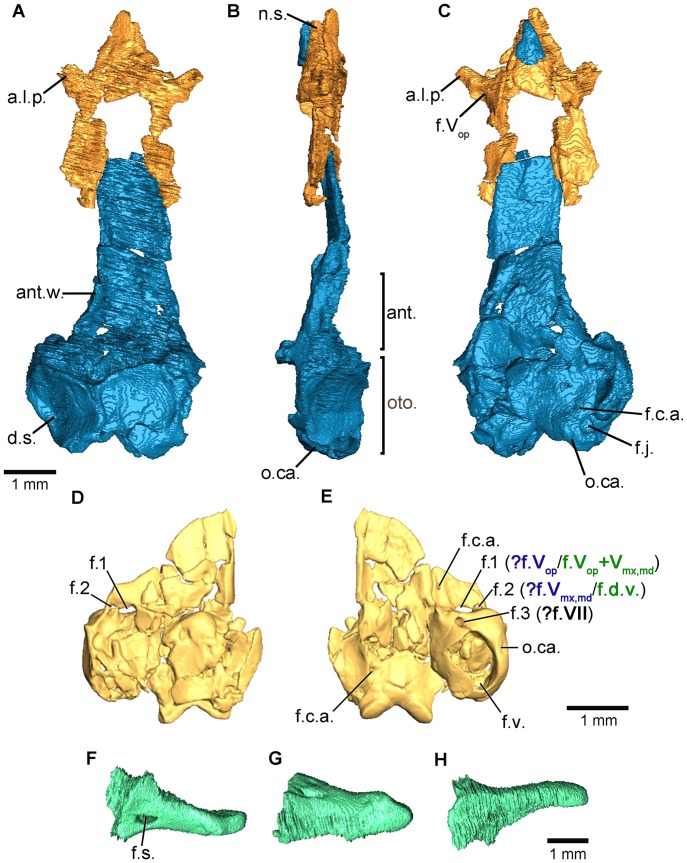
The braincase and middle ear ossicle of *Eocaecilia micropodia* as revealed by µCT. A–C, three-dimensional digitally segmented braincase of the holotype (MNA V8066) in dorsal, left lateral and ventral views, respectively, with the sphenethmoid (orange) and the os basale (blue). D–E, surface renderings of an isolated braincase referred to *E. micropodia* (MNA V8063) in dorsal and ventral views, respectively. The alternative hypotheses of foramen identity are depicted in blue and green. F–H, three-dimensional digitally segmented middle ear ossicle of *E. micropodia* (MNA V8066) in dorsal, left lateral, and ventral views respectively. This element is termed the stapes-quadrate by Jenkins et al. [14] because of the hypothesized fusion of these two elements. Abbreviations: ant., antotic region; ant.w., antotic wall; a.l.p., anterolateral process; d.s., dorsal surface of the otic capsule; f.c.a., foramen for the carotid artery; f.d.v., foramen for a dorsal vein; f.j., jugular foramen; f.s., stapedial foramen; f.v., fenestra vestibuli; f.1, foramen 1 (see text for interpretation); f.2, foramen 2 (see text for interpretation), f.V_mx,md_, foramen for the maxillary plus mandibular trunk of the trigeminal nerve; f.V_op_, foramen for the ophthalmic branch of the trigeminal nerve; n.s., nasal septum; oto., otic-occipital complex; o.ca., otic capsule.

The holotype of *Eocaecilia micropodia* (MNA V8066) preserves a virtually complete skull and articulated lower jaws (Figure 1). Micro-computed tomography (µCT) confirms that the braincase of *Eocaecilia micropodia* consists of two bones (Figure 2A–C), similar in general morphology to the two composite bones comprising the braincase in extant caecilians, i.e., the sphenethmoid and the os basale [32]. The sphenethmoid of *E. micropodia* has relatively long lateral walls that make up more than 50% of the preserved length of the sphenethmoid (Figure 2A). The dorsal sutural surfaces for connecting to the dermal skull roof appear to be narrow, but it is difficult to determine whether the dorsal margins of the lateral walls are broken or are preserved in their natural state.

The anterolateral corners of the main body of the sphenethmoid bear robust, ossified, anterolaterally-directed processes (a.l.p.; Figure 2A,C) that strongly resemble the condition seen in many extant caecilians [28]. The data acquired from µCT show these to be longer than depicted in the reconstruction of Jenkins et al. [14]. Jenkins et al. [14] identified two foramina located within each anterolateral process of the sphenethmoid in *E. micropodia*. The first, a large foramen located in the base of the process, was justifiably identified as transmitting the ophthalmic branch of the trigeminal nerve (f.V_op_; Figure 2C), based on observations of consistency of this arrangement in extant caecilians [27]. An additional foramen located dorsal to that for the ophthalmic branch of the trigeminal nerve was identified as a foramen transmitting a vessel. This foramen is not visible in MNA V8066, but is present in MNA V8059. The interpreted identity of this second foramen as a vascular foramen is reasonable given the similarity with the condition seen in several extant caecilians [14]. An alternative interpretation may be that the superficial ramus of the facial nerve splits from an anastomosis with the ophthalmic branch of the trigeminal nerve prior to entering the anterolateral process of the sphenethmoid, resulting in a second foramen, as seen in *Geotrypetes seraphini* and *Herpele squalostoma* [27], [28].

A dorsomedial process (mesethmoid of some authors [15]) like that of extant caecilians was not seen in the µCT data. If such a process were present, but not preserved in any specimen, the positional relationship between the sphenethmoid and dermal skull indicates it would have been located just deep to the frontals. Because there is no fontanelle between the frontals, dorsal exposure of the sphenethmoid on the skull roof is precluded.

**Figure 3:**
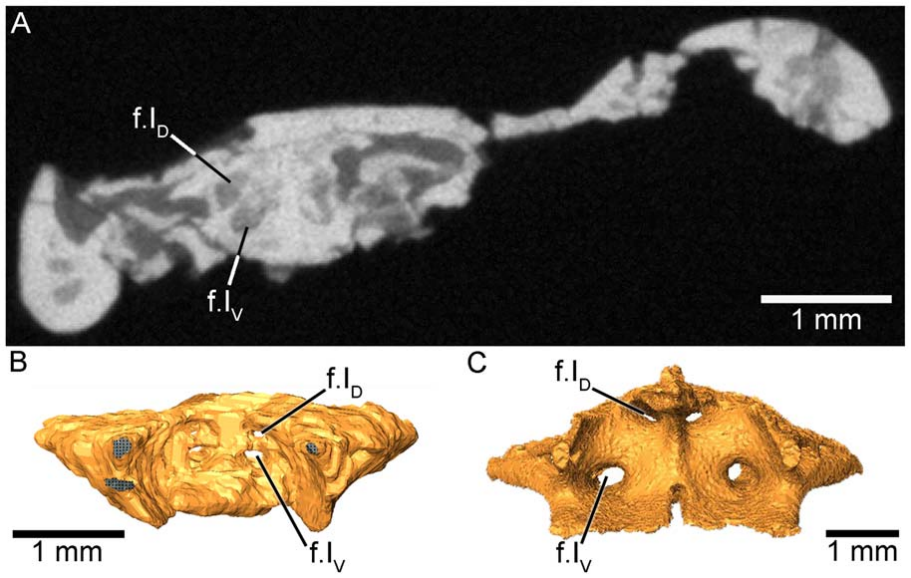
Identification of paired dorsal and ventral foramina, serving the likely transmission of the paired trunks of the olfactory nerve, similar to the condition seen in extant caecilians. A, µCT image of a transverse section through the level of the anterior sphenethmoid in *E. micropodia* (MNA V8066), showing the location of dorsal and ventral foramina in the anterior wall of the sphenethmoid. B, posterior view of the three-dimensionally segmented sphenethmoid of *E. micropodia*, further showing the locations of the anterior foramina interpreted here as those serving the dorsal and ventral trunks of the olfactory nerve. C, posterior view of the three-dimensionally segmented sphenethmoid of the extant caecilian *Dermophis mexicanus* (UMMZ 219030), showing the location of comparable foramina known to transmit the trunks of the olfactory nerve [14]. Abbreviation: f.I_D_, foramen for the dorsal branch of the olfactory nerve; f.I_V_, foramen for the ventral branch of the olfactory nerve.

Anteriorly the sphenethmoid is completed by a wall that separates the nasal capsules from the brain cavity, as seen in extant caecilians. The µCT reconstruction reveals that the anterior wall is perforated by two pairs of foramina (f.I_D_ and f.I_V_; Figure 3A, B). The paired dorsal and ventral anterior foramina lie close to the midline of the sphenethmoid. These are interpreted here, based on similarity with the condition seen in extant caecilians (Figure 3C), as serving the olfactory nerve. The paired dorsal and ventral foramina suggest the olfactory nerve was bifurcated into dorsal and ventral trunks upon emergence from the brain, as it is in all extant caecilians [27], [33].

The nasal region is represented by an ossified short, robust nasal septum (n.s.; Figure 2B). It is not known whether the tip of the nasal septum is complete or broken. A broad dorsal sutural surface extends anteriorly from the main body of the sphenethmoid to over the nasal septum. It tapers sharply in width towards the tip (Figure 2A). The ventral margin of the nasal septum is narrow. There is no evidence of sola nasi in *E. micropodia*.

The antotic walls (or pleurosphenoid of some authors [23]) are long and subparallel in dorsal view in *E. micropodia* (ant.w.; Figure 2A). The dorsal margins of the antotic walls appear incomplete and therefore the nature of the dorsal sutural surface is unknown. The anterior margins of the antotic walls may also be incomplete in the holotype of *E. micropodia,* but Jenkins et al. [14] describe a concave anterior margin, likely corresponding to the posterior margin of the optic foramen, as in extant caecilians [28].

Much of the antotic region is poorly preserved in the holotype specimen (ant.; Figure 2B). However, the right side of the specimen consisting of the isolated braincase (MNA V8063) yields some additional information (Figure 2D, E). There are three larger foramina present. Foramen one (f.1; Figure 2E) is located dorsally, just anterior to the otic capsule. A slightly larger second foramen is present ventral to this one (f.2; Figure 2E). The third foramen (f.3, Figure 2E) is located ventral and slightly posterior to the first two, along the anteroventral margin of the otic capsule. The latter two (f.2 and f.3; Figure 2E) were identified by Jenkins et al. [14] as those pertaining to the trigeminal and facial nerves, respectively. The location of foramen one occurs in a similar location to the foramen seen among the extant forms to transmit a dorsal vein [27], which may be the case for *E. micropodia* as well. An alternative interpretation is one in which the two trunks of the trigeminal nerve, the maxillary plus mandibular trunk and the ophthalmic trunk, exit the braincase from foramen one and two, and the facial nerve from foramen three (Figure 2E). An additional foramen serving a dorsal vein may have been present but is not preserved by the broken dorsal portion of the wall. Given the relative positions and sizes of the foramina, in comparison to those seen in extant caecilians, the latter hypothesis is preferred here and is most similar to the condition seen in rhinatrematid, dermophiid, and siphonopid caecilians (Pattern 1 of Maddin [27]). A small, fourth foramen is present in the antotic region, located anterior to the three just described (f.c.a.; Figure 2E). A groove runs anteriorly from this foramen, and it was reasonably identified as that for the anterior branch of the internal carotid artery [14]. A similarly located foramen serves this function in extant caecilians.

The otic-occipital complex of the braincase (oto.; Figure 2B) is very similar in morphology to that of most extant caecilians. The dorsal surface of the complex forms the roof of the posteriormost portion of the brain cavity (d.s.; Figure 2A). The surface on each side tapers in width towards the midline. In *E. micropodia*, however, the surface remains relatively broad in comparison with that of most extant caecilians. The dorsal surface of the otic-occipital complex is slightly concave on either side. It is unclear whether the postparietals (plesiomorphic, discrete ossifications posterior to the parietals) contribute to the occipital surface, or if the entire occipital surface is composed only of the braincase, similar to the condition seen in basal caecilians such as *Rhinatrema bivittatum* and *Epicrionops bicolor* [28]. It is also unclear from the µCT data whether the anterior margin of the dorsal surface bears a thin sutural surface that receives the postparietals in *E. micropodia*, similarly to that which receives the parietals in most extant caecilians, where postparietals are presumed lost. The lateral surface of the otic capsule is occupied by a large, laterally facing *fenestra vestibuli*. The occipital condyles are continuous in profile with the posterior margin of the otic-occipital complex in the holotype specimen (o.ca.; Figure 2B and E). In the isolated braincase the condyles appear somewhat more posteriorly protuberant (Figure 2D), but the incomplete posterior margins of the otic capsules may give an inaccurate appearance of condyle protrusion. The condyles, however, are small in comparison to those of most extant caecilians. A jugular foramen is present between the otic capsule and the occipital condyle (Figure 2E).

The anterior portion of the floor of the *os basale* is triangular in outline (Figure 2C), similar to the condition seen in *Rhinatrema bivittatum*. The floor extends to reach the tip of the preserved portion of the nasal septum. Jenkins et al. [14] described the presence of three depressions in the floor of the brain cavity: one posterior depression and a pair of anterior depressions. The posterior depression likely corresponds to that seen in extant caecilians for the hypophysis of the brain, and the anterior pair of depressions likely corresponds to those associated with the large cerebral hemispheres of the brain, also seen in extant caecilians [27]. Well-developed, wing-like basicranial articulations are absent from *E. micropodia*. Also absent is a well-defined muscle attachment site on the ventral surface of the otic-occipital complex (Figure 2) that is often present in extant caecilians.

The structures of the middle ear are intimately associated with the braincase of tetrapods and are therefore given consideration here. *Eocaecilia micropodia* possesses an atypical configuration of bones in the middle ear region, making interpretation of the homology of elements difficult. A large element applied to the lateral surface of the otic capsule with a discrete foramen and jaw articulation was interpreted by Jenkins et al. [14] as a fusion of the stapes and quadrate (i.e., the stapes-quadrate; Figure 2F–H). An additional element resembling a small disc-like bone, closely associated with the fenestra vestibuli and stapes-quadrate, was interpreted as an operculum [14]. An alternative hypothesis is one in which the element identified as the operculum is the stapes, and the larger element consists of the quadrate alone [14], [16].

### Phylogenetic Analysis of Eocaecilia Micropodia

**Figure 4:**
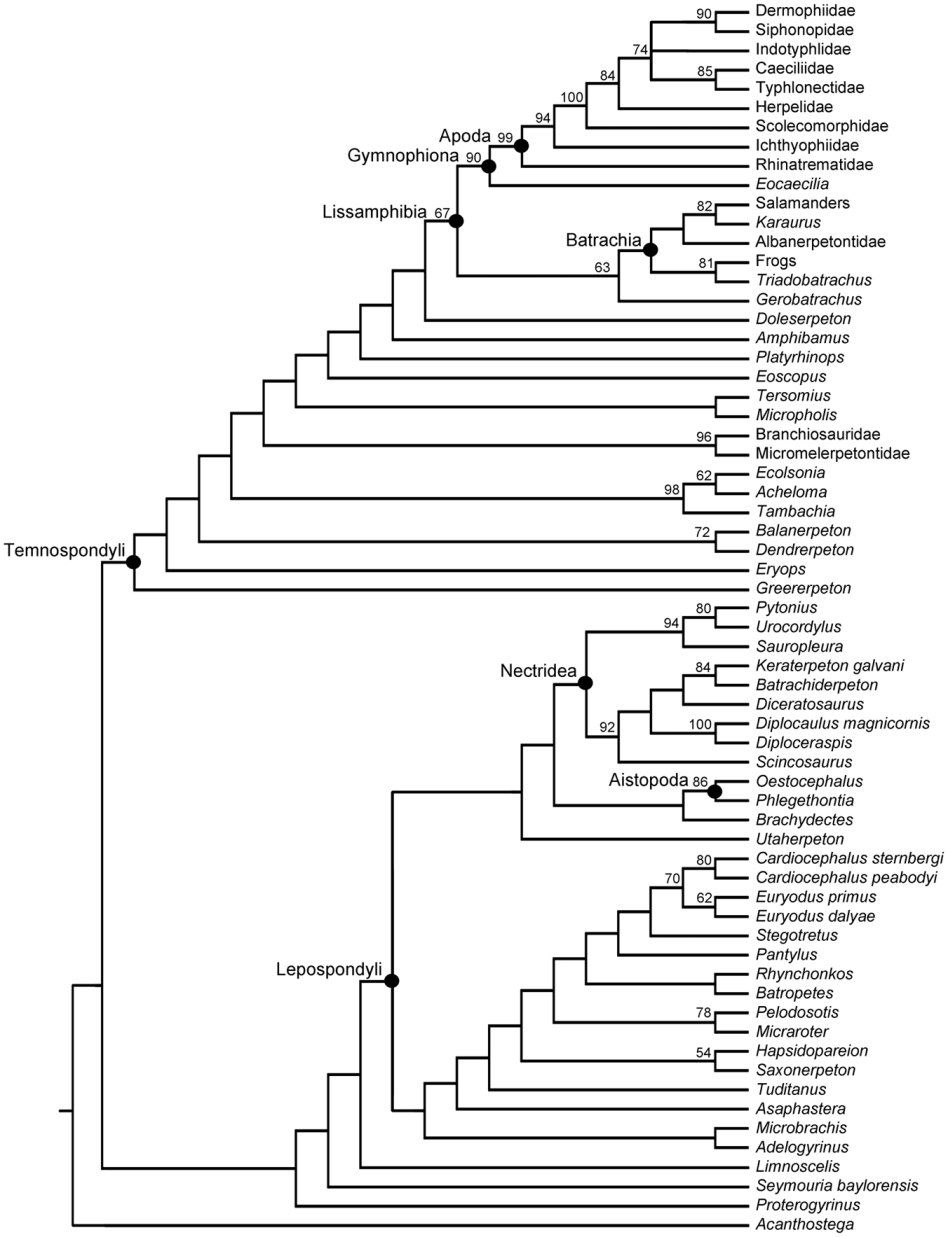
The 50% majority-rule consensus tree of the 34 most parsimonious trees obtained in the parsimony analysis (1450 steps). Numbers above nodes represent bootstrap values greater than 50% (values below 50% not shown). Within the temnospondyl lineage a monophyletic Lissamphibia was obtained. *Eocaecilia micropodia* was recovered on the stem of extant caecilians (Apoda) and together this clade (Gymnophiona) formed the sister taxon to the clade of Batrachia plus *Gerobatrachus*. The sister taxon to Lissamphibia in this hypothesis is *Doleserpeton*.

The analysis of the combined matrix developed here results in thirty-four most parsimonious trees, each 1,450 steps in length (consistency index equals 0.352, retention index equals 0.654, rescaled consistency index equals 0.230, and the homoplasy index equals 0.719). The 50% majority rule consensus tree is well resolved (Figure 4); however, overall support for the tree is low. Of the sixty-one recovered nodes, less than half (twenty-seven) have bootstrap values above 50%, and only eighteen are above 75%.

Within the temnospondyl lineage, the analysis retrieves a monophyletic Lissamphibia (bootstrap support of 67% and 27 synapomorphies) with the sister taxon being *Doleserpeton*. Of the 27 synapomorphies of Lissamphibia, 16 are unambiguous (A5, A45, A49, A65, A84, A87, A91, A97, A100, A126, A129, A130, A134, A136, A140, A141), three of which are reversals. A monophyletic Batrachia is obtained, with *Gerobatrachus* located on its stem (bootstrap support of 63%). *Eocaecilia micropodia* is robustly placed on the stem of the clade consisting of extant caecilians (bootstrap support of 90%). This hypothesis is supported by 37 synapomorphies (29 cranial, 8 postcranial). Extant caecilians plus *E. micropodia* form the sister taxon to Batrachia plus *Gerobatrachus*.

Within the lepospondyl lineage, the analysis retrieves a paraphyletic ‘Microsauria’ due to the placement of *Utaherpeton* as more closely related to the remaining lepospondyls than to the other microsaurs. A monophyletic Nectridea is retrieved as the sister taxon to a clade comprising the lysorophian taxon *Brachydectes* plus a monophyletic Aïstopoda.

### Results of the Ancestral Character State Reconstructions

The results of the ancestral character state reconstructions derived by the three reconstruction methods (maximum parsimony, maximum likelihood, and Bayesian Inference) are largely congruent in their estimation for the state at the base of Apoda (Table 1). The maximum parsimony (MP) approach resolved twenty-eight of thirty-four characters as state 0 for the base of extant caecilians, four characters as state 1 (state 0 in Maddin et al. [28]), and the other two characters fail to be resolved between state 0 and alternative states (Table 1). Under the maximum likelihood (ML) approach all but four of thirty-four characters were resolved as state 0 for the base of extant caecilians, the other four being state 1, as in the MP approach. Twenty-three of the thirty-four characters were resolved with likelihoods of greater than 0.70. The Bayesian inference (BI) approach, similarly to the ML approach, resolved all but four of thirty-four characters as state 0 for the base of extant caecilians, the other four being state 1 as in the MP approach, twenty-seven of which with significant support (Bayes factor greater than 2). Of the 21 braincase characters that *E. micropodia* could be scored for, 18 characters bear an equivalent state to the condition inferred to be plesiomorphic for Apoda (Table 1).

**Table 1:** Ancestral character state reconstructions of the 34 braincase characters from Maddin et al. [28] for the condition at the base of extant caecilians and a comparison with the condition seen in *Eocaecilia micropodia*.

Character	MP	ML	ML	BI	BI	BI	*Eocaecilia*
number	State	State	Probability	State	Bayes Factor	Support	State
79	1	1	0.997	1	10.51	>10	2*
80	0	0	0.542	0	10.51	>10	0
81	1	1	0.997	1	10.51	>10	?
82	0	0	0.865	0	0.53	0–2	?
83	0	0	0.999	0	10.51	>10	?
84	0	0	0.613	0	1.54	0–2	?
85	0	0	0.999	0	5.79	>5	1*
86	0	0	0.9	0	4.08	>2	0
87	0,1	0	0.511	0	4.08	>2	?
88	0	0	0.999	0	0.07	0–2	0
89	0	0	0.533	0	0.52	0–2	0
90	1	1	0.999	1	21.35	>10	?
91	0	0	0.997	0	21.35	>10	0
92	0	0	0.742	0	21.35	>10	0
93	1	1	0.678	1	21.35	>10	1
94	0	0	0.986	0	21.35	>10	0
95	0	0	0.999	0	21.35	>10	0
96	0	0	0.633	0	22.42	>10	0
97	0	0	0.998	0	22.42	>10	2*
98	0	0	0.801	0	22.42	>10	?
99	0	0	0.999	0	22.42	>10	0
100	0	0	0.518	0	22.42	>10	0
101	0	0	0.999	0	22.42	>10	0
102	0	0	0.716	0	5.4	>5	0
103	0	0	0.652	0	4.39	>2	0
104	0,3,4	0	0.643	0	4.39	>2	0
105	0	0	0.981	0	0.91	0–2	?
106	0	0	0.696	0	0.91	0–2	0
107	0	0	0.999	0	0.91	0–2	0
108	0	0	0.999	0	16.02	>10	–
109	0	0	0.959	0	16.02	>10	–
110	0	0	0.991	0	16.02	>10	?
111	0	0	0.999	0	16.02	>10	–
112	0	0	0.999	0	16.02	>10	?

**BI support**: 0–2, weak support; >2, support; >5 strong support; >10 very strong support.

States with asterisk for *E. micropodia* are those that conflict with the hypothesized ancestral state of extant caecilians.

## Discussion

### New Morphological Features

The application of micro-computed tomography (µCT) to the skull of *Eocaecilia micropodia* revealed the presence of several new features of the braincase previously inaccessible through traditional methods. The µCT data reveals that the sphenethmoid is significantly better ossified than previously thought. This includes a completely ossified anterior wall separating the brain and nasal cavities, a somewhat elongate and ossified nasal septum, and expanded anterolateral corners. A similar ossified process is present in many extant caecilians, where the process is completed distally by cartilage that is applied to the medial surface of the maxillopalatine, or the prefrontal in the case of Scolecomorphidae [28]. In the case of taxa lacking this ossified process, an entirely cartilaginous process is present (e.g., *Caecilia, Geotrypetes*, *Idiocranium* [27], [28]). The process creates a bridge or strut-like structure between the dermal skull and the braincase. The presence of cartilage with its compressible properties suggests this process may have some functional role as a compression sink to alleviate stress placed on the skull when engaging in head-first burrowing activity. A potentially analogous bracing mechanism is utilized in amphisbaenians, wherein broad flanges of the orbitosphenoid contact the medial wall of the frontals [34]; however, this appears to be a bone-bone contact and there is no cartilage involved in the contact. Among the taxa examined here, this ossified anterolateral process pierced by the ophthalmic branch of the trigeminal nerve is interpreted as a synapomorphy of *E. micropodia* and extant caecilians.

An important observation made here was that of the potentially paired dorsal and ventral foramina located within the anterior wall of the sphenethmoid. Rarely is the anterior wall of the sphenethmoid ossified in taxa, but when it is (e.g., the aïstopod *Phlegethontia*), a single pair of foramina pierce the bone for transmission of the olfactory nerve (or tracts) to the nasal capsule [35]. To our knowledge, caecilians are unique among tetrapods in possessing an olfactory nerve that splits into distinct dorsal and ventral trunks upon emergence from the brain. Histological examination reveals that the dorsal trunk of the olfactory nerve supplies the nasal capsule, and the ventral trunk supplies both the nasal capsule and the laterally positioned vomeronasal organ (or Jacobson’s organ [36], [37]). The vomeronasal organ of caecilians is considered to be elaborated in comparison to that of frogs and salamanders [38], [39], and its innervation leads to a correspondingly elaborated region of the brain (i.e., the large, morphologically differentiated accessory olfactory bulbs [39]). Chemoreception in the caecilian vomeronasal organ is thought to be facilitated largely by the tentacle [38], a uniquely caecilian organ derived from co-opted structures associated with the eye [40]. A distinct foramen for the ventral trunk of the olfactory nerve in *E. micropodia* suggests that a caecilian-like neurological configuration, in which a well-developed ventral nerve trunk emerges separately from an enlarged accessory olfactory bulb, was already in place. This in turn suggests the vomeronasal organ was already undergoing elaboration. The identification of an slit-like opening adjacent to the eye (similar to that for the tentacle of basal extant caecilians) in *E. micropodia* may represent such an early pathway for increased reception by means of a tentacle or tentacle precursor. However, the small size of the putative opening in *E. micropodia* suggests the reorganization of the neurological components preceded elaboration of the tentacle and may have been a necessary first step in the evolution of this highly specialized structure. Together, the additional ventral foramina in the sphenethmoid and their potential soft tissue correlates represent another new synapomorphy of *E. micropodia* and extant caecilians.

### Implications of the Phylogenetic Context and Ancestral Character State Reconstructions

The phylogenetic analysis conducted here confidently places *Eocaecilia micropodia* on the stem of the clade consisting of extant caecilians. This result supports the hypothesis of Jenkins and Walsh [12] and Jenkins et al. [14] that *E. micropodia* is a gymnophionan. The broad sampling of temnospondyl taxa and the high support for *E. micropodia* plus crown caecilians refute the suggestion that *E. micropodia* may occupy a different position within Dissorophoidea [19].

Additionally, *E. micropodia* plus extant caecilians are recovered as the sister taxon to a temnospondyl Batrachia (plus *Gerobatrachus*). This is in contrast to the hypothesis previously yielded by analysis of the matrix of Anderson et al. [18], wherein caecilians nested among lepospondyls, creating a polyphyletic “Lissamphibia”. The recovery of *E. micropodia* plus extant caecilians as the sister taxon to batrachians plus frogs and salamanders is supported by 27 synapomorphies, 26 of which are characters common to the matrix of Anderson et al. [18]. This suggests the movement of *E. micropodia* to the sister taxon position to the temnospondyl batrachians was sufficient to reveal a whole suite of previously obscured potential synapomorphies of Lissamphibia (see results). Additionally, the hypothesized pattern of relationships supports the growing consensus of temnospondyl-derived lissamphibian monophyly. The next most closely related taxon to Lissamphibia is *Doleserpeton*, an animal considered highly relevant to lissamphibian origins since its discovery [41], [42].

Of the thirty-four braincase and stapes characters, *E. micropodia* is scored for twenty-one. Three of the missing entries (scored ‘not applicable’) are attributed to the unusual morphology and uncertain identity of the middle ear ossicle(s) in *E. micropodia*, and as such the remaining discussion of the ancestral character state reconstructions (ACSR) concerns the braincase only. The ACSRs conducted here are in accordance with the hypothesis that the braincase of *E. micropodia* is representative of the plesiomorphic condition of extant caecilians, with few exceptions. The first character state that conflicts with the inferred plesiomorphic condition of extant caecilians is the short length of the ossified nasal septum in *E*. *micropodia* (character M79). It was noted in the description that the nasal septum may not be complete, and so it may be that it is actually much longer. However, there is little space for the septum to continue anteriorly, and so it is likely that the short condition is accurate for *E. micropodia*. In this way, *E. micropodia* is convergent with typhlonectid caecilians. The second character state in *E. micropodia* that conflicts with the inferred plesiomorphic condition of extant caecilians is the long lateral walls of the sphenethmoid (character M85), which in *E. micropodia* appears to be convergent with scolecomorphid caecilians. Finally, the third character state that differs from the inferred plesiomorphic condition is the relatively broad dorsal exposure of the os basale in *E. micropodia* (character M97). This broad exposure may be more like the condition seen in advanced dissorophoids like *Doleserpeton*, suggesting extant caecilians are derived in their possession of a narrower exposure.

In all other regards, the morphology of the braincase of *E. micropodia* appears to estimate the plesiomorphic condition seen in extant caecilians. This is in contrast to the dermal skull, in which the morphology of *E. micropodia* is fairly distinct from the condition observed in extant caecilians, especially when compared to those considered to be basal members of the group. This is consistent with the hypothesis that evolutionary changes in the braincase take place at a slower rate than the dermal skull and that it is therefore more likely to retain phylogenetically informative characteristics in the braincase than the other regions of the skull [24]–[28].

### Implications for Cranial Evolution in Lissamphibia

Given the phylogenetic topology supported here, the morphology of the skull of *E. micropodia* is interpreted as representative of the condition of the skull of the last common ancestor of Lissamphibia. The robust, closed temporal condition of the skull of *E. micropodia* has been interpreted as autapomorphic for *E. micropodia* and convergently derived, with that of advanced extant caecilians, associated with a fossorial lifestyle. However, the closed temporal region in extant caecilians has recently been shown to not result in improved performance of the skull during burrowing activity [43], leaving the identification of the source(s) of this transformation in extant caecilians open for interpretation. When compared to the next most closely related taxon to lissamphibians, *Doleserpeton*, much of the skull of *E. micropodia* can be interpreted as symplesiomorphic for Dissorophoidea [23]. This includes the closed temporal region and the pattern of the skull roof bones, including the presence of many of the dermal bones that are absent in extant lissamphibians [42].

**Figure 5:**
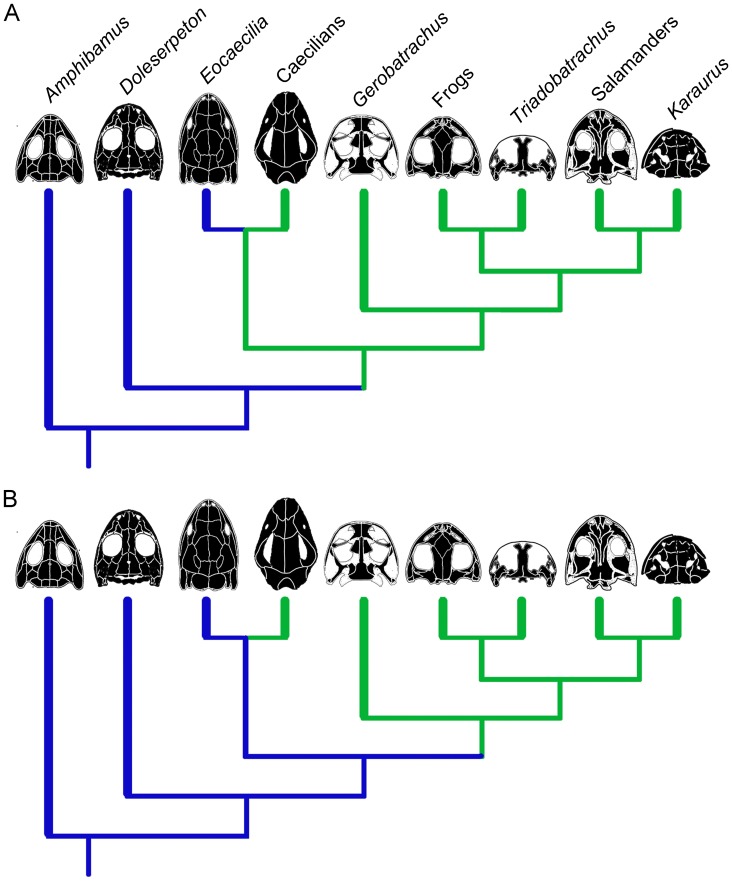
Alternative hypotheses for the evolution of the open temporal region in extant lissamphibians. A, Scenario in which the open condition is homologous for lissamphibians, and *Eocaecilia micropodia* is a homoplastic reversal to the condition present in *Doleserpeton*. B, Scenario in which the open condition is a homoplastic convergence between extant caecilians and batrachians plus *Gerobatrachus*. This hypothesis is favoured here given the occurrence of a similar condition in lysorophian lepospondyls. Illustrations modified from: *Amphibamus* [51]; *Doleserpeton* [42]; *Eocaecilia* [14]; caecilians [52]; *Gerobatrachus* [18]; frogs [21]; *Triadobatrachus* [53]; salamanders [21]; *Karaurus* [54].

However, the condition of the skull shared between basal extant caecilians and basal extant frogs and salamanders warrants consideration of alternative hypotheses for the evolution of the skull of lissamphibians [19], [20], [22]. Under one scenario, the condition expressed in basal extant lissamphibians is considered homologous. This would then represent the condition present in the last common ancestor of the three groups, and the condition expressed in *E. micropodia* becomes a homoplastic reversal to the condition present in dissorophoid temnospondyls (Figure 5A). Alternatively, the condition expressed in *Doleserpeton* (and *E. micropodia*) is representative of the plesiomorphic condition of Lissamphibia, and the condition expressed in extant caecilians and batrachians plus *Gerobatrachus* represents homoplastic convergence (or parallelism) between the two clades (Figure 5B). Both hypotheses are equally parsimonious (each requiring 2 steps). Examination of the tetrapod fossil record reveals at least one additional occurrence of an open, zygokrotaphic-like condition in the non-amniote lepospondyl *Phlegethontia* (Fig. 4; [35]). This may be seen as evidence to support a greater likelihood of the second hypothesis, i.e., evolving an open skull condition multiple times in tetrapods [14].

The close evolutionary proximity of two out of the three homoplastic instances of zygokrotaphy in non-amniote tetrapods supports the idea that a homologous predisposition to a zygokrotaphic condition may be present in members of Lissamphibia. Such a predisposition might be a shared ground pattern of the skull and a constrained ontogenetic trajectory. Subsequent evolutionary developmental perturbation in both the caecilian and batrachian lineages, such as those caused by various heterochronic events, could result in similar end point morphologies and therefore, give rise to this homoplastic distribution via parallelism [44]. Paedomorphosis via neoteny or progenesis, and miniaturization are examples of such mechanisms, and those which have been attributed to the origin of the lissamphibian form in the past (e.g., [41], [45]–[48]). Although these mechanisms have mostly been explored in the evolution of the batrachian lineage, several features suggest similar mechanisms may have been operating in the evolution of the caecilian lineage as well. Such features include the loss of some/all circumorbital bones [49], reduction in the cranial base [28], [48], aspects of the palate [50], and ontogeny of the temporal region [19]. The scant fossil record of caecilians precludes further refinement of evolutionary developmental mechanism at this time.

We conclude, based on the data presented here, that the plesiomorphic condition of Gymnophiona (stem-based definition) is stegokrotaphic and that the plesiomorphic condition of Apoda (node-based definition) is zygokrotaphic. We emphasize that the stegokrotaphic condition exhibited by *E. micropodia* (all roofing elements present) is fundamentally different from that which is expressed by apodans (many roofing elements absent [14], [19], [20]), and that neither pattern may be associated with improved mechanics during burrowing. Such features are more likely to be the streamlined skull outline, reduced orbits, and overall body form.

## Supporting Information

Table S1 **List of character state changes made in the current analysis, from those of Anderson et al.** [**18**] **.**
(DOCX)

Text S1 **List of characters used in the current phylogenetic analysis.** Characters A1–A219 correspond to those from Anderson et al. [18] and characters M1–M112 correspond to those from Maddin et al. [28], where M1–M78 are from Wilkinson [55]. New characters are denoted by N1–N5. Changes to character definitions are indicated in bold parentheses where they occur. Characters excluded are in grey with the reason for exclusion indicated in bold parentheses where they occur.
(DOCX)

Dataset S1 **Nexus file of the character-taxon matrix used in the current study.**
(NEX)
