# Supplementary material for: The Braincase of Eocaecilia micropodia (Lissamphibia, Gymnophiona) and the Origin of Caecilians
Source: PLoS One. 2012 Dec 5;7(12):e50743. doi: 10.1371/journal.pone.0050743 (PMC3515621; doi:10.1371/journal.pone.0050743)
Supplement: Table S1 — List of character state changes made in the current analysis, from those of Anderson et al. [18] . (DOCX) [file pone.0050743.s001.docx]

Table S1. List of character state changes made in the current analysis, from those of Anderson et al. [18].

| **Character number** | **Modification** | **Character number** | **Modification** |
| --- | --- | --- | --- |
| A1 | Albanerpetontidae ? ⇒3  Frog ? ⇒0/1  Salamander ?⇒0/1 | A87 | *Eocaecilia* 3⇒1  Frog 0⇒1  *Gerobatrachus* 2⇒1  Salamander ? ⇒1  *Triadobatrachus* 0⇒1 |
| A2 | Albanerpetontidae ? ⇒2 | A90 | Frog ? ⇒1  Salamander ? ⇒1 |
| A3 | Salamander ? ⇒1 | A94 | Frog 1⇒3  Salamander ? ⇒3  *Gerobatrachus* 1⇒3 |
| A5 | *Gerobatrachus* 0⇒1 | A96 | *Gerobatrachus* ? ⇒1 |
| A6 | *Gerobatrachus* 0⇒ – | A97 | *Eocaecilia* 0⇒1 |
| A11 | *Gerobatrachus* 1⇒0 | A98 | *Eocaecilia* 1⇒0 |
| A18 | *Triadobatrachus* 0⇒1  Salamander – ⇒1 | A115 | *Eocaecilia* 0⇒1  Frog 1⇒2  *Gerobatrachus* 1⇒2  Salamander 1⇒2  *Triadobatrachus* 1⇒2 |
| A20 | Salamander 1⇒ – | A132 | *Eocaecilia* 1⇒2 |
| A21 | Frog ? ⇒0 | A146 | Salamander ? ⇒0 |
| A25 | Frog – ⇒0  Salamander – ⇒0 | A149 | *Gerobatrachus* 1⇒2 |
| A29 | *Gerobatrachus* 0⇒1 | A165 | *Gerobatrachus* 0⇒? |
| A33 | Salamander 2⇒1 | A166 | *Eocaecilia* 1⇒0 |
| A34 | Salamander ? ⇒1 | A169 | *Eocaecilia* 2⇒1  *Triadobatrachus* ? ⇒0 |
| A45 | *Gerobatrachus* 0⇒? | A203 | Salamander 0⇒1 |
| A59 | *Eocaecilia* ? ⇒0 | A205 | Albanerpetontidae ? ⇒0  *Triadobatrachus* 1⇒0 |
| A74 | Salamander ? ⇒0 | A207 | Albanerpetontidae 0⇒?  *Triadobatrachus* 0⇒? |
| A78 | *Gerobatrachus* 0⇒? | A208 | *Eocaecilia* 2⇒0 |
| A86 | Albanerpetontidae -⇒1  Salamander ? ⇒1 | A209 | Albanerpetontidae ? ⇒1  *Eocaecilia* ? ⇒1  Salamander 2⇒1 |
