# Supplementary material for: The Braincase of Eocaecilia micropodia (Lissamphibia, Gymnophiona) and the Origin of Caecilians
Source: PLoS One. 2012 Dec 5;7(12):e50743. doi: 10.1371/journal.pone.0050743 (PMC3515621; doi:10.1371/journal.pone.0050743)
Supplement: Text S1 — List of characters used in the current phylogenetic analysis. Characters A1–A219 correspond to those from Anderson et al. [18] and characters M1–M112 correspond to those from Maddin et al. [28], where M1–M78 are from Wilkinson [55]. New characters are denoted by N1–N5. Changes to character definitions are indicated in bold parentheses where they occur. Characters excluded are in grey with the reason for exclusion indicated in bold parentheses where they occur. (DOCX) [file pone.0050743.s002.docx]

Text S2. List of characters used in the current phylogenetic analysis. Characters A1-A219 correspond to those from Anderson et al. [18] and characters M1-M112 correspond to those from Maddin et al. [28], where M1-M78 are from Wilkinson [55]. New characters are denoted by N1-N5. Changes to character definitions are indicated in bold parentheses where they occur. Characters excluded are in grey with the reason for exclusion indicated in bold parentheses where they occur.

A1. Basal skull length: (0) >70mm, (1) 50-70mm, (2) 30-50mm, (3) <30mm.

A2. Skull to trunk: (0)>=0.45, (1) 0.30-0.45, (2) 0.20-0.29, (3) 0.20.

A3. Skull proportions: (0) longer than wide, (1) wider than long.

A4. Intertemporal: (0) present, (1) absent.

A5. Supratemporal: (0) present, (1) absent.

A6. Supratemporal exposure on occiput: (0) absent, (1) present.

A7. Tabular-postorbital contact: (0) absent, (1) present.

A8. Tabular-postfrontal contact: (0) absent, (1) present.

A9. Postfrontal shape: (0) broadly quadrangular, (1) falciform.

A10. Squamosal-tabular contact: (0) absent, (1) present, (2) fused.

A11. Squamosal-temporal area: (0) weakly overlapping, (1) sutural.

A12. Lacrimal-prefrontal suture: (0) simple butt joint, (1) interdigitating, (2) prefrontal broadly underplates lacrimal.

A13. Lacrimal: (0) present, (1) absent.

A14. Lacrimal-naris contact: (0) present, (1) absent.

A15. Lacrimal contribution to orbit: (0) absent, (1) present.

A16. Lacrimal orbital processes: (0) only ventral present, (1) dorsal and ventral present, (2) neither present.

A17. Lacrimal-jugal contact: (0) present, (1) absent.

A18. Quadratojugal: (0) present, (1) absent.

A19. Quadratojugal-Jugal contact: (0) present, (1) absent.

A20. Quadratojugal-maxilla contact: (0) present, (1) absent.

A21. Frontals: (0) paired along midline, (1) fused.

A22. Frontal contribution to orbit: (0) no, (1) yes.

A23. Anterior laterally flaring frontals: (0) absent, (1) present.

A24. Nasals: (0) present, (1) absent.

A25. Nasal flange: (0) absent, (1) present.

A26. Alary processes of premax: (0) absent, (1) present.

A27. Internarial fontanelle: (0) absent, (1) present.

A28. Septomaxilla: (0) ossified, (1) unossified.

A29. Prefrontal contributes to external naris: (0) distant from, (1) near, (2) present.

A30. External naris in dorsal view: (0) exposed, (1) not exposed.

A31. External naris shape: (0) circular, (1) posteriorly extended, along lacrimal-prefrontal suture, (2) posteriorly extended, excavation of lacrimalonly.

A32. Dorsal exposure of premaxilla: (0) broad, (1) narrow, (2) none.

A33. Dorsal shape of skull: (0) triangular, (1) diamond, (2) rounded.

A34. Posterior skull margin: (0) concave, (1) straight, (2) convex, (3) undulating.

A35. Snout: (0) blunt, (1) pointed.

A36. Snout: (0) short, (1) long.

A37. Quadrate internal flange of squamosal: (0) absent, (1) present.

A38. Otic notch: (0) present, (1) absent.

A39. Large otic notch approaching orbit: (0) absent, (1) intermediate, (2) close.

A40. Otic notch: (0) open posteriorly, (1) closed posteriorly.

A41. Semilunar flange of supratemporal: (0) absent, (1) present.

A42. Supratympanic flange: (0) absent, (1) present "trematopid-like", (2) present "dissorophid-like".

A43. Supratympanic shelf: (0) absent, (1) present.

A44. Raised orbital rim: (0) absent, (1) present.

A45. Postorbital: (0) present, (1) absent.

A46. Jugal-postorbital interfingered processes: (0) absent, (1) present.

A47. Postorbital contribution to orbital margin: (0) present, (1) absent.

A48. Shape of postorbital: (0) irregular trapezoid, (1) triangular, apex caudal.

A49. Palpebral ossifications: (0) absent, (1) mosaic of bone plates present in orbit.

A50. Parietal-postorbital contact: (0) absent, (1) present.

A51. Parietal-squamosal contact: (0) absent, (1) present.

A52. Parietal-tabular contact: (0) absent, (1) present.

A53. Postparietals: (0) paired, (1) fused, (2) absent.

A54. Parietal foramen: (0) present, (1) absent.

A55. Postparietals: (0) moderate, (1) large.

A56. Postparietal-squamosal contact: (0) absent, (1) present.

A57. Postparietal length: (0) large, quadrangular, (1) abbreviated anteroposteriorly, elongate lateral rectangle.

A58. Squamosal-jugal contact: (0) present, (1) absent.

A59. Tabular: (0) present, (1) absent.

A60. Posterolateral projection from lateral margin of tabular above squamosal embayment: (0) absent, (1) present.

A61. Tabular horns: (0) absent, (1) present.

A62. Tabular horns: (0) parallel or slightly divergent, (1) widely divergent.

A63. Squamosal forms base of tabular horn: (0) absent, (1) present.

A64. Lateral line canal grooves: (0) present, (1) absent.

A65. Dermal sculpturing: (0) circular pits, (1) shallow ridges and grooves, (2) little to none.

A66. Premaxilla anterior margin: (0) vertical, (1) overturned.

A67. Maxilla contributes to orbit: (0) no, (1) yes.

A68. Maxilla into external naris: (0) present, (1) absent.

A69. Maxilla forms entire ventral naris: (0) absent, (1) present.

A70. Maxilla: (0) longer than palatine, (1) shorter than palatine.

A71. Marginal teeth: (0) vertical, (1) turned medially.

A72. Marginal teeth largest anterior: (0) absent, (1) present.

A73. Marginal teeth: (0) pointed pegs, (1) blunt pegs, (2) large cones.

A74. Number of premax teeth: (0) >=10, (1) 5-9, (2) <5.

A75. Number of max teeth: (0) >=30, (1) 20-29, (2) <20.

A76. Teeth laterally compressed: (0) no, (1) yes.

A77. Enlarged teeth mid-toothrow: (0) absent, (1) present.

A78. Teeth: (0) simple points, (1) multiple cusps.

A79. Multiple Cusp Orientation: (0) Labio-lingual, (1) anterio-posterior.

A80. Enamel fluting: (0) absent, (1) present.

A81. Labyrinthine in-folding: (0) present, (1) absent.

A82. Occipital profile: (0) low and wide, (1) high and wide, (2) high and narrow.

A83. Supraoccipital: (0) absent, (1) present.

A84. Occipital condyle: (0) concave, (1) convex.

A85. Occipital condyle: (0) single, with basioccipital, (1) double.

A86. Jugular foramen: (0) between opistotic and exoccipital, (1) through exoccipital.

A87. Jaw articulation: (0) posterior to occiput, (1) even with occiput, (2) anterior to occiput, (3) far anterior (>20% BSL).

A88. Internal nares: (0) widely separated, (1) narrowly separated.

A89. Palatal teeth: (0) present, (1) absent.

A90. Palatine teeth: (0) single pit-pairs, (1) multiple in rows, (2) multiple random.

A91. LEP: (0) absent, (1) present.

A92. Anterior palatine: (0) short anteromedial process articulating with vomer at choana, (1) long anteromedial process, more medial than lateral, (2) palatine absent.

A93. Vomerine teeth: (0) present, (1) absent.

A94. Vomer teeth: (0) single pit pairs, (1) multiple in rows, (2) multiple random, (3) single row, tiny teeth **(new state added here)**.

A95. Intervomerine depression: (0) absent, (1) present.

A96. Intervomerine rostral fenestration: (0) absent, (1) present.

A97. Denticles on pterygoid: (0) present, (1) absent.

A98. Teeth on pterygoid: (0) absent, (1) present.

A99. Tooth pedicely: (0) absent, (1) present.

A100. Denticles on vomers: (0) present, (1) absent.

A101. Denticles on palatines: (0) present, (1) absent.

A102. Denticles on parasphenoid: (0) present, (1) absent.

A103. Palatal teeth: (0) larger than marginals, (1) equal to marginal’s, (2) smaller than marginals.

A104. Parasphenoid: (0) medial of stapes, (1) under footplate of stapes.

A105. Parasphenoid basal plate: (0) roughly quadrangular, basipterygoid articulations narrowly spaced, (1) rectangular laterally, anteroposteriorly narow, basipterygoid articulations distant.

A106. Cultriform process contact with vomer: (0) narrow, (1) broad.

A107. Basicranial articulation: (0) loose, (1) sutured or fused.

A108. Stapes: (0) perforated columella, (1) imperforate columella, (2) no columella.

A109. Stapes orientation: (0) lateral, towards quadrate, (1) dorsal, towards squamosal embayment, elongate columella.

A110. Footplate of stapes: (0) oval, (1) round, (2) palmate.

A111. Dorsal process of stapes: (0) absent, (1) present.

A112. Accessory ossicle in middle ear: (0) absent, (1) present.

A113. Pleurosphenoid: (0) unossified, (1) ossified.

A114. Sphenethmoid: (0) ossified, (1) unossified.

A115. Interpterygoid vacuities: (0) narrow (closed), (1) wide, (2) extremely wide **(new state added here)**, (3) fused at midline.

A116. Pterygoids contact anteriorly: (0) present, (1) absent.

A117. Pterygoid-exoccipital contact: (0) absent, (1) present.

A118. Pterygoid-palatine suture: (0) present, (1) absent.

A119. Pterygoid-vomer contact: (0) present, (1) absent.

A120. Lateral process of pterygoid contribution to posttemporal fossa: (0) absent, (1) present.

A121. Ectopterygoid: (0) present with fang-pit pair, (1) present lacking fang-pit pair, (2) absent.

A122. Ectopterygoid-palatine width: (0) wider than maxilla, (1) narrower than maxilla.

A123. Pharyngeobranchial pouches: (0) absent, (1) present.

A124. Dentary: (0) long, (1) short.

A125. Dentary forms coronoid process: (0) absent, (1) present.

A126. Surangular: (0) normal, (1) reduced, (2) absent.

A127. Angular: (0) narrow, (1) deep.

A128. Number of splenials: (0) 2, (1) 1, (2) 0.

A129. Splenial exposed laterally: (0) present, (1) absent.

A130. Meckelian fossae: (0) 2 or more, (1) 1, (2) 0.

A131. Ventral border of Meckel’s fossa: (0) splenial, (1) angular.

A132. Retroarticular process: (0) absent, (1) present, small, (2) present, long **(character state added)**.

A133. Retroarticular process: (0) straight, (1) hooked.

A134. Articulation to tooth row: (0) above, (1) equal, (2) below.

A135. Angular extends to (lat view): (0) posterior tooth row, (1) middle of tooth row.

A136. Number of coronoids: (0) 3, (1) 2, (2) 1, (3) 0.

A137. Coronoid teeth: (0) present, (1) absent.

A138. Coronoid teeth: (0) larger than marginals, (1) equal to marginals, (2) smaller than marginals.

A139. Symphysis: (0) dentary and splenial, (1) dentary alone.

A140. Jaw sculpture: (0) present, (1) absent.

A141. Ossified hyoids: (0) present, (1) absent.

A142. Gill osteoderms: (0) absent, (1) present, noninterdigitating, (2) toothed, intergiditating rakers.

A143. Parahyoid: (0) absent, (1) present.

A144. Number of accessory articulation: (0) 0, (1) 1, (2) 2 or more.

A145. Number of presacrals: (0) 25-35, (1) 20-24, (2) >35, (3) <20.

A146. Vertebral development: (0) arches, then centra, (1) centra and arches simultaneously.

A147. Caudal processes between depression: (0) absent, (1) present.

A148. Trunk intercentra: (0) present, (1) absent.

A149. Trunk neural arch to centrum: (0) loosely articulated, (1) sutured, (2) fused.

A150. Base of neural spine: (0) equal to or wider than haemal, (1) smaller than haemal spine.

A151. Height of neural spines: (0) even, (1) alternating.

A152. Dorsal neural spine: (0) narrow and smooth, (1) laterally broad and sculpted.

A153. Neural spine shape (lat): (0) ant-post sides parallel (rect), (1) non-parallel (triangular).

A154. Neural spine lateral suface: (0) smooth, (1) crenulated.

A155. Pleurocentra: (0) paired rhachitomous, (1) closely approaching ventrally, (2) fused, dominant weight bearing element.

A156. Haemal arches: (0) present, (1) absent.

A157. Haemal arches: (0) loosely articulated intercentrum, (1) fused to mid-length of centrum.

A158. Haemal arches: (0) longer than or equal to neurals, (1) shorter than neurals.

A159. Haemal accessory articulations: (0) none, (1) one, (2) two.

A160. Haemal arch shape: (0) non-parallel (triangular), (1) parallel (rectangular).

A161. Tail: (0) tapers, (1) deep with sudden end.

A162. Tail: (0) elongate, equal to or exceeding trunk and skull length, (1) forshortened, markedly shorter than trunk.

A163. Trunk arches: (0) paired, (1) fused.

A164. Spinal nerve foramina: (0) absent, (1) present.

A165. Extended transverse processes: (0) absent, (1) present.

A166. Transverse process: (0) on arch pedicle, (1) on centrum.

A167. Atlas-axis intercentra: (0) present, (1) absent.

A168. Atlas anterior centrum: (0) same size as posterior, (1) laterally expanded.

A169. Atlas centrum: (0) multipartite, (1) single notochordal, (2) single odontoid.

A170. Atlas neural arch: (0) loosely articulated, (1) sutured to centrum, (2) fused to centrum.

A171. Atlas parapophyses: (0) on centrum, (1) on transverse process, (2) absent.

A172. Atlas neural arch: (0) paired, (1) sutured at midline, (2) fused at midline.

A173. Atlas accessory articulation: (0) absent, (1) zygosphene, (2) zygantra.

A174. Proatlantes: (0) present, (1) absent.

A175. Second cervical arch: (0) expanded to more posterior, (1) equal to more posterior, (2) shorter than more posterior.

A176. Atlas ribs: (0) one pair, (1) two pairs, (2) absent.

A177. Cervical rib distal shape: (0) spatulate, (1) pointed.

A178. Ribs anterior to sacrum: (0) short, (1) long.

A179. Ribs: (0) elongated and sometimes curved, (1) straight, (2) short, simple rod.

A180. Costal process at rib head: (0) absent, (1) present.

A181. Number of sacrals: (0) 1, (1) 2, (2) 3.

A182. Sacral parapophysis: (0) on centrum, (1) on transverse process.

A183. Number pairs of caudal ribs: (0) 5 or more, (1) 4, (2) 3, (3) 2 or fewer.

A184. Interclavicle posterior stem: (0) no or short, (1) long.

A185. Interclavicle posterior stem: (0) wide, (1) narrow.

A186. Interclavicle: (0) diamond shaped, (1) t-shaped.

A187. Interclavicle anterior plate: (0) broad, (1) narrow.

A188. Interclavicle shape-diamond: (0) broad diamond, (1) narrow diamond.

A189. Interclavicle anterior fimbrati: (0) present, (1) absent.

A190. Interclavicle sculpture: (0) present, (1) absent.

A191. Cleithrum head: (0) aligned along anterior rim of scapula, (1) posterodorsally enlarged head wrapping around dorsal scapula.

A192. Cleithrum head: (0) dorsally greatly expanded, much wider than shaft, (1) simple rod without or slight dorsal expansion.

A193. Cleithrum: (0) ossified, (1) unossified.

A194. Cleithrum: (0) rounded or pointed dorsally, (1) t- or y-shaped.

A195. Proximal clavicle blades: (0) widely separate, (1) articulate medially, (2) interdigitate.

A196. Supraglenoid foramen: (0) present, (1) absent.

A197. Number coracoid foramina: (0) 0, (1) 1, (2) 2.

A198. Scapulocoracoid ossification: (0) both, (1) scapula only, (2) absent.

A199. Entepicondylar foramen: (0) present, (1) absent.

A200. Torsion in humerus: (0) absent, (1) less than 80 degrees, (2) more than 80 degrees.

A201. Deltapectoral crest: (0) weak, (1) intermediate, (2) prominent.

A202. Supinator process: (0) absent, (1) present.

A203. Humerus length: (0) long (>4 trunk centra), (1) short.

A204. Radius:humerus: (0) >=0.7, (1) 0.5-0.7, (2) <0.5.

A205. Olecranon process: (0) unossified, (1) ossified.

A206. Carpals: (0) fully or partially ossified, (1) unossified.

A207. Basale commune: (0) absent, (1) present.

A208. Number of manual digits: (0) 5 or more, (1) 4, (2) 3.

A209. Pelvis: (0) fused, (1) sutured, (2) poorly ossified.

A210. Anteriorly inclined ilium: (0) absent (1) present.

A211. Illiac blade: (0) 2 dorsal processes, (1) narrowly bifurcate, (2) single blade.

A212. Internal trochanter-articulation: (0) distinct, (1) continuous.

A213. Femoral shaft: (0) robust, (1) slender.

A214. Femur: (0) long, (1) short.

A215. Tarsals: (0) ossified, (1) unossified.

A216. Elongate tibiale and fibulare: (0) absent (1) present.

A217. Number of distal tarsals: (0) 6, (1) 5 or fewer.

A218. Astragulus: (0) absent, (1) present.

A219. Number of pedal digits: (0) 5 or more, (1) 4 or less.

1. True tail (postcloacal vertebrae and annuli): (0) present, (1) absent. [T1]
2. Tertiary annuli: (0) absent, (1) present. [T2]
3. Mouth: (0) terminal, (1) subterminal, (2) strongly subterminal. [T3]
4. Nasopremaxilla: (0) separate, (1) fused. [T4]
5. Septomaxilla: (0) present, (1) absent. [T5]
6. Prefrontals: (0) present, (1) absent. [T6]
7. Postfrontals: (0) present, (1) absent. [T7]
8. Squamosal-frontal contact: (0) contact, (1) no contact. [T8]
9. Squamosal notch – os basale process: (0) absent, (1) present. [T9] (**Autapomorphic for Rhinatrematidae)**
10. Zygokrotaphy: (0) stegokrotaphy including roofing bones like tabulars and supratemporals **(state added here)**, (1) zygokrotaphy with muscles approaching skull midline, (2) stegokrotaphy in the absence of roofing bones such as tabulars and supratemporals, (3) zygokrotaphy, but muscle does not extend through temporal fossa. [T10]

M11. Parasphenoid: (0) parallel walls, (1) converge anteriorly. [T13]

M12. Orbitosphenoid: (0) vertical, (1) oblique. [T14] (**Autapomorphic for Scolecomorphidae)**

M13. Quadrate-maxilla separated by: (0) pterygoid, (1) small pterygoid and pterygoid process of quadrate, (2) by pterygoid process of quadrate only (pterygoid absent). [T15a]

M14. Mediopalatinal canal: (0) expanded (pterygoid and pterygoid process absent), (1) not expanded. [T15b] (**Autapomorphic Scolecomorphidae)**

M15. Basipterygoid process: (0) absent, (1) weakly developed, (2) well developed. [T16] (**Redundant with A107)**

M16. Stapes: (0) perforate, (1) imperforate. [T17] (**Redundant with A108)**

M17. Quadrate-maxillopalatine: (0) contact laterally, (1) do not contact laterally. [T18]

M18. Retroarticular process: (0) short and straight, (1) long and recurved. [T19] **Redundant with A133**

M19. Glossal skeleton: (0) posterior glossal skeleton reduced in size, ceratobranchial (cb) 4 absent, (1) cb 3 and 4 fused and a little expanded, (2) cb 3 and 4 fused and much expanded. [T20a]

M20. Ceratobranchials: (0) 3 and 4 fused enclosing larynx, (1) not. [T20b]

1. Fibres of m. interhyoideus anterior: (0) insert on ceratohyal, (1) do not. [T22]

M22. M. interhyoideus posterior: (0) in one bundle, (1) two bundles. [T24]

M23. M. interhyoideus posterior: (0) predominantly oblique, (1) predominantly horizontal. [T25]

M24. M. depressor mandibulae: (0) predominantly vertical, (1) predominantly longitudinal. [T27]

M25. Orbit: (0) open, (1) closed. [T28]

M26. Tentacle: (0) absent (state added here), (1) present adjacent to eye, (2) between eye and naris, (3) beneath naris. [T29]

M27. Phallodeum: (0) absent **(state added here)**, (1) present and aspinous, (2) spinous. [T30]

M28. Vent: (0) longitudinal, (1) transverse or circular. [T31]

M29. Splenial teeth: (0) present, (1) absent. [T32]

M30. Choanal openings: (0) small, (1) large. [T33]

M31. Narial plugs: (0) absent, (1) present. [T35]

M32. Sphenethmoid: (0) covered dorsally, (1) exposed dorsally. [T36]

M33. Prevomerine teeth: (0) without medial diastema, (1) with medial diastema. [T38]

M34. Choanae: (0) not completely encircled by maxillopalatine, (1) completely encircled. [T39]

M35. Teeth: (0) some or all premaxillary teeth small, (1) all large. [T40] (**Autapomorphic for Scolecomorphidae)**

M36. Development: (0) oviparous, (1) viviparous. [T42]

M37. Larval stage: (0) present, (1) absent, direct development. [T43]

M38. Atria: (0) not divided externally, (1) divided externally. [T44] (**Autapomorphic for Ichthyophiidae)**

M39. Anterior pericardial space: (0) short and small, (1) long and extensive. [T45] (**Autapomorphic for Ichthyophiidae)**

M40. Posterior internal flexures in m. rectus laterals: (0) less than two, (1) two. [T46] (**Autapomorphic for Ichthyophiidae)**

M41. Internal flexures on m. subvertebralis: (0) none, (1) one. [T47] (**Autapomorphic for Ichthyophiidae)**

M42. Tracheal lung: (0) absent, (1) present. [T48] (**Autapomorphic for Ichthyophiidae)**

M43. Anterior annuli: (0) orthoplicate, (1) angulate. [T49] (**Autapomorphic for Ichthyophiidae)**

M44. Paired m. rectus laterales: (0) meet mid-dorsally, (1) separated. [T50]

M45. Anterior internal flexures in m. rectus laterales: (0) one, (1) none. [T51]

M46. ‘Myosepta’ in m. obliquus externus superficialis: (0) well developed, (1) dorsal only, (2) absent. [T52]

M47. Origin of ventral part of m. subvertebralis: (0) midcentrum, (1) subvertebral. [T53]

M48. Terminal keel: (0) absent, (1) present. [T54]

M49. Annular scales: (0) scales, secondary annuli and segmented body present, (1) absent. [T55]

M50. M. interhyoideus posterior: (0) short, (1) elongate. [T56] (**Autapomorphic for Scolecomorphidae)**

M51. Anterior dentary teeth: (0) bicuspid, (1) monocuspid. [T57]

M52. Vomeropalatine tooth row: (0) semicircular, (1) at an angle, anteriorly. [T58]

M53. Rectus externus present (0), or not (1). [E1.1] **(Lacks phylogenetic signal)**

M54. Rectus internus present (0), or not (1). [E1.2] **(Lacks phylogenetic signal)**

M55. Rectus superior present (0), or not (1). [E1.3] **(Lacks phylogenetic signal)**

M56. Rectus inferior present (0), or not (1). [E1.4] **(Lacks phylogenetic signal)**

M57. Superior oblique present (0), or absent (1). [E1.5] **(Lacks phylogenetic signal)**

M58. Inferior oblique present (0), or not (1). [E1.6] **(Lacks phylogenetic signal)**

M59. Eye in orbital chamber (0), or riding on the tentacle (1). [E2] (**Autapomorphic for Scolecomorphidae)**

M60. Optic nerve well developed (0), attenuate or absent (1). [E3] **(Lacks phylogenetic signal)**

M61. Vitreous body present (0), or absent (1). [E4] **(Lacks phylogenetic signal)**

M62. More than 5000 retinal cells (0), or less (1). [E6] **(Lacks phylogenetic signal)**

M63. Lens present (0), rudimentary (1), or absent (2). [E10] **(Lacks phylogenetic signal)**

M64. Stapes present (0), or absent (1). [A1] (**Autapomorphic for Scolecomorphidae)**

M65. Lagena sensory epithelium present (0), or absent (1). [A2]

M66. Well-developed lagenal recess with a median recess at the saccular orifice and associated basilar papilla present (0), or lagenal recess reduced and basilar papilla absent (1). [A3]

M67. Basilar papilla on limbic tissue (0), or not (1). [A5]

M68. Papilla neglecta near utriculus-sacculus foramen (0), or not (1). [A9]

M69. Number of hair cells in papilla neglecta less than half (0), approximately equal to (1), or more than twice (2) the number in the papilla amphibiorum. [A10] (**Autapomorphic for Typhlonectidae (state 1) and Scolecomorphidae (state 2))**

M70. Spinal 1 contributes to hypoglossal (0), or not (1). [H1.1]

M71. Spinal 3 contributes to hypoglossal (0), or not (1). [H1.2]

M72. Vagal ramus X-1 contributes to hypoglossal (0), or not (1). [H1.3]

M73. Occipital nerve present (0), or not (1). [H2]

M74. Hypoglossal in the tongue bifurcated (0), straight with branches along its length (1), branches at tip only (2). [H6]

M75. Undivided (0), slightly divided (1), or divided (2) main nasal cavity. [O1]

M76. Respiratory epithelium posterolaterally (0), or throughout (1) the nasal cavity. [O3] **Autapomorphic for Typhlonectidae**

M77. Vomeronasal organ mediolateral (0), mediolateral with lateral projection (1), or lateral (2). [O4]

M78. Vomeronasal organ moderate (0), or large (1). [O6] (**Autapomorphic for Typhlonectidae)**

1. Nasal septum: (0) not ossified (new state added here), (1) 20-35% of the total skull length, (2) less than 20% of the total skull length.
2. Nasal septum: (0) tall at tip, weakly tapered in height, (1) short at tip, strongly tapered in height.
3. Dorsomedial process of the sphenethmoid: (0) absent (new state added here), (1) 11% or more of the total skull length, (2) 10% or less of the total skull length.
4. Dorsomedial process of the sphenethmoid: (0) thin and rod-like, (1) thick and broad.
5. Dorsal sutural surface of the sphenethmoid: (0) narrow or weakly tapered, (1) broad and triangular and strongly tapered.
6. Sola nasi: (0) absent, (1) present and connected to the nasal septum ventrally, (2) present, rod-like or tubular.
7. Length of the lateral walls of the sphenethmoid: (0) 14% or less than the total skull length, (1) 15% or greater of the total skull length.
8. Floor of sphenethmoid: (0) deeply incised and U-shaped in outline, (1) shallowly incised with broad coverage of os basale, (2) deeply incised and U-shaped, but with midline extension.
9. Thin sheet of bone anterior and ventral to ventral olfactory foramina: (0) absent, (1) present.
10. Dorsal olfactory nerve foramina: (0) foramina enclosed within sphenethmoid, (1) troughs that are open dorsally instead of foramina.
11. Ventral olfactory foramina: (0) directly ventral to the dorsal foramina, (1) located laterally relative to the dorsal foramina, away from midline.

M90. Groove for palatal ramus of the facial nerve: (0) absent (new state added here), (1) in the lateral walls of the main body of the sphenethmoid, (2) in the ventral surface of the main body of the sphenethmoid.

M91. Anterolateral foramen: (0) complete laterally, (1) incomplete laterally.

M92. Anterior extent of the floor of the os basale: (0) extends to encroach within the region of the anterior half of nasal septum, (1) does not encroach on the nasal septum, (2) extends to encroach within the region of the posterior half of the nasal septum.

M93. Margin of the floor of the anterior portion of the parasphenoid (definition changed here): (0) narrow (new state added here), (1) broad, tapers continuously to a point, (2) rounded anteriorly, (3) markedly tapered, resulting in bottleneck appearance, (4) rod-like distal tip.

M94. Parasphenoid margins (definition changed here): (0) absence of lateral constriction posterior to basicranial articulations, (1) weak constriction posterior to basicranial articulations present, (2) strong constriction posterior to basicranial articulations, reaching to the carotid foramina.

M95. Quadrate articular fossa dorsal to antotic foramina: (0) absent, (1) present.

M96. Angle between antotic walls and skull roof portion of os basale when viewed laterally: (0) smooth curve, angle greater than 90˚, (1) abrupt, right angle.

M97. Skull roof exposure of the os basale: (0) moderate exposure at the dorsal midline, (1) little to no exposure near the dorsal midline, (2) very wide exposure near the dorsal midline.

M98. Occipital surface: (0) completely made up by the os basale, (1) incompletely made up by the os basale, receives contribution from parietal.

M99. Fenestra vestibuli: (0) thickened ventral margin of fenestra vestibuli absent, (1) present.

M100. Fenestra vestibuli: (0) large, subcircular, (1) anteroposteriorly elongate, oval, (2) very small, subcircular.

M101. Fenestra vestibuli: (0) opening directed laterally, (1) opening directed anteriorly.

M102. Occipital condyles: (0) in lateral view condyles are continuous with the outline of the otic-occipital complex to weakly protruding, (1) condyles protrude well beyond posterior limit of the otic-occipital complex.

M103. Ventral muscle attachment site: (0) no ventral projections, (1) deep, wing-like processes at lateral margins of the site.

M104. Antotic foramina: (0) Pattern 1, (1) Pattern 2, (2) Pattern 3, (3) Pattern 4, (4) Pattern 5, (5) Pattern 6, (6) Pattern 7, (7) Pattern 8. [from Chapter Maddin, 2011]

M105. Canal for palatal ramus of the facial nerve in antotic region: (0) absent, (1) present.

M106. Facial nerve and ventral vein foramina: (0) facial nerve and ventral vein exit through separate foramina, (1) both exit through a common foramen.

M107. Footplate of stapes: (0) fills the fenestra vestibuli, (1) much smaller than the fenestra vestibuli.

M108. Orientation of columellar process of the stapes: (0) horizontal, (1) anterodorsally oriented.

M109. Ridge on lateral margin of columellar process: (0) absent, (1) present.

M110. Fossa on anterior margin of footplate: (0) present and small, (1) present and very large.

M111. Columellar process: (0) no ridge present, (1) ridge on distal columellar process.

M112. Shape of optic foramen: (0) large, subcircular opening, (1) narrow and slit-like opening, (2) oblique anterior margin in the sphenethmoid, (3) oblique posterior margin in the os basale.

N1. Limbs: (0) present, (1) absent.

N2. Skull table: (0) rectangular, (1) T-shaped.

N3. Operculum ossicle (0) absent, (1) present.

N4. Anterior wall of sphenethmoid: (0) unossified, (1) ossified, one pair of foramina, (2) ossified, two pairs of foramina (dorsal and ventral).

N5. Anterolateral process of sphenethmoid: (0) absent, (1) present.
